# Differentiation of spontaneously contracting cardiomyocytes from non-virally reprogrammed human amniotic fluid stem cells

**DOI:** 10.1371/journal.pone.0177824

**Published:** 2017-05-17

**Authors:** Aaron J. Velasquez-Mao, Christopher J. M. Tsao, Madeline N. Monroe, Xavier Legras, Beatrice Bissig-Choisat, Karl-Dimiter Bissig, Rodrigo Ruano, Jeffrey G. Jacot

**Affiliations:** 1 Department of Bioengineering, Rice University, Houston, TX, United States of America; 2 Department of Molecular and Cellular Biology, Center for Cell and Gene Therapy, Stem Cells and Regenerative Medicine Center, Baylor College of Medicine, Houston, TX, United States of America; 3 Department of Obstetrics and Gynecology, Maternal Fetal Medicine Texas Children’s Hospital, Houston, TX, United States of America; 4 Congenital Heart Surgery Service, Texas Children’s Hospital, Houston, TX, United States of America; 5 University of Colorado Denver, Department of Bioengineering, Aurora, CO, United States of America; University of Texas at Austin Dell Medical School, UNITED STATES

## Abstract

Congenital heart defects are the most common birth defect. The limiting factor in tissue engineering repair strategies is an autologous source of functional cardiomyocytes. Amniotic fluid contains an ideal cell source for prenatal harvest and use in correction of congenital heart defects. This study aims to investigate the potential of amniotic fluid-derived stem cells (AFSC) to undergo non-viral reprogramming into induced pluripotent stem cells (iPSC) followed by growth-factor-free differentiation into functional cardiomyocytes. AFSC from human second trimester amniotic fluid were transfected by non-viral vesicle fusion with modified mRNA of *OCT4*, *KLF4*, *SOX2*, *LIN28*, *cMYC* and nuclear *GFP* over 18 days, then differentiated using inhibitors of GSK3 followed 48 hours later by inhibition of WNT. AFSC-derived iPSC had high expression of OCT4, NANOG, TRA-1-60, and TRA-1-81 after 18 days of mRNA transfection and formed teratomas containing mesodermal, ectodermal, and endodermal germ layers in immunodeficient mice. By Day 30 of cardiomyocyte differentiation, cells contracted spontaneously, expressed connexin 43 and β-myosin heavy chain organized in sarcomeric banding patterns, expressed cardiac troponin T and β-myosin heavy chain, showed upregulation of NKX2.5, ISL-1 and cardiac troponin T with downregulation of POU5F1, and displayed calcium and voltage transients similar to those in developing cardiomyocytes. These results demonstrate that cells from human amniotic fluid can be differentiated through a pluripotent state into functional cardiomyocytes.

## Introduction

Congenital heart defects (CHD) are the most common birth defects and the leading cause of infant death in the United States [1]. Autologously derived contractile cardiac cells can be applied to patches for structural defect repair [2], engineered heart tissue[3], cells for cardiomyoplasty [4], and gene editing correction of specific defects[5]. With 80% of CHDs diagnosed in the second trimester [6], amniotic fluid presents an ideal source for autologous cells for use in neonatal CHD treatment [4, 7].

Amniotic fluid stem cells (AFSC) are broadly multipotent, but do not directly differentiate into contractile cardiomyocytes (CM). Specifically, AFSC express mesenchymal stem cell markers (CD29, CD44, CD90, and CD105), certain pluripotent markers (SOX2), and are capable of differentiating into all three germ layers[8]. While attempts at direct cardiac differentiation have shown gene and protein level similarities (GATA4, Nkx2.5, α-actinin, cTnT), resulting cells ultimately lack contractility[8, 9].

Induced pluripotent stem cells (iPSC) can be differentiated into force-generating CM [3, 4, 10], and studies show that iPSC can be generated from AFSC [11, 12]. However, no study has investigated the transformation of AFSC into CM using non-virally attained iPSC as an intermediary.

The objectives of this study were to test whether AFSC can be reprogrammed to iPSC by mRNA delivery and whether non-virally attained AFSC-iPSC are capable of cardiac differentiation. Reprogrammed AFSC were evaluated for pluripotency by protein expression and teratoma formation. CM derived from AFSC-iPSC were evaluated for expression of cardiac genes and proteins, membrane potential fluctuation, calcium handling, and contractile function.

## Materials and methods

### AFSC culture isolation and expansion

AFSC were isolated based on previously published methods from our group[8, 13]. Primary human amniotic fluid was obtained from patients in their second trimester undergoing planned amnioreduction as part of a therapeutic treatment for twin-twin transfusion syndrome (TTTS). Amniotic fluid was centrifuged at 1200 rpm for 10 min, and collected cells were plated at 2500 cells/cm2 on standard plastic Petri dishes and cultured in a modified α-Minimum Essential Media: 63% αMEM (Invitrogen, Carlsbad, CA), 18% Chang Basal Medium (Irvine Scientific, Santa Ana, CA), 2% Chang C supplement (Irvine Scientific), 15% fetal bovine serum (PAA Laboratories, Dartmouth, MA), and GlutaMAX (Invitrogen) at 37°C and 5% CO2 in a humidified environment. Media was changed every two to three days, and cells were passaged at 60–70% confluence. At the first passage, a subpopulation of progenitor cells was isolated through fluorescence-activated cell sorting for expression of the membrane receptor CD117/c-kit (BD Biosciences, Bedford, MA). Cell colonies were detached into single cells (Accutase; Sigma-Aldrich, St. Louis, MO; 37°C, 10 min), and c-kit+ cells were collected using a Dako MoFlo sterile cell sorter. All studies of primary human cells were approved by the Institutional Review Boards of both Baylor College of Medicine and Rice University, and subjects gave informed consent.

### iPSC generation and culture

AFSC were transfected with mRNA to generate an iPS state using the Stemgent mRNA Reprogramming System (Lexington, MA)[14]. Briefly, frozen c-kit+ passage 2 AFSC, were thawed and plated onto 100mm petri dishes. The cells were allowed to expand to 80% confluency and then plated in 6 well plates containing a feeder layer of mitomycin-treated newborn human foreskin fibroblasts (NuFF, Stemgent, Inc., Cambridge, MA). After attachment, transfection of the AFSC was carried out by exposure to reprogramming factors (Oct4, Klf4, Sox2, c-Myc) for 4 hours each day for 18 days. Briefly, AFSC were plated on a feeder layer of NuFF in Pluriton Reprogramming Medium (Stemgent) supplemented with 4ng/mL bFGF (Stemgent) and B18R recombinant protein (eBioscience, Inc., San Diego, CA). AFSC were exposed for 4 hours per day to an mRNA cocktail comprised of OCT4, SOX2, KLF4, c-Myc, LIN28, and nGFP (TriLink Biotechnologies Inc., San Diego, CA) complexed with Lipofectamine (RNAiMAX, Thermo Fisher Scientific, Carlsbad, CA) for 18 consecutive days. At the end of the 18-day transfection, cell colonies were selected based on morphology and the pluripotency expression marker TRA-1-81. Each well yielded approximately 10 iPSC colonies per well that were each 1–2 mm in diameter. Colonies were then continuously split and passaged every 5–7 days onto mouse embryonic fibroblast feeder (MEF, GlobalStem, Inc., Rockville, MD) on treated 6-well plates, and maintained using CDF12 medium as described by Warren et al 18: DMEM/F-12 (Invitrogen, Carlsbad, CA), knockout serum replacement (Life Technologies, Carlsbad, CA), non-essential amino acids (Life Technologies), Glutamax (Life Technologies), 2-mercaptoethanol (Gibco, Carlsbad, CA), 20ng/mL bFGF (Stemgent), and 1x penicillin-streptomycin. Cells were passaged using 0.1% collagenase type 4 (Worthington Biochemical Corp., Lakewood, NJ) in DMEM/F-12 medium (Invitrogen). Each well of the new passage was seeded with 4 colonies that were broken up physically by scraping with a 10mL pipet and gentle trituration, and yielded approximately 30–40 new colonies that grew to 2–5 mm in diameter before the next passage.

### Teratoma formation

Animal experiments were approved by the Institutional Animal Care and Use Committee (IACUC) of Baylor College of Medicine and conformed to the Guide for the Care and Use of Laboratory Animals as stated by the NIH. In order to characterize the AFSC-iPSC self-renewal and pluripotency properties, a teratoma study was conducted to assess their ability to differentiate into derivatives of the three embryonic germ layers. This work was done by the Bisseg lab at Baylor College of Medicine. Briefly, approximately 1x10^6 AFSC-iPSC were injected subcutaneously into SCID mice (8–12 weeks of age) and monitored for 8–10 weeks for teratoma formation. Teratoma evaluation was done by histology and H&E staining.

### Immunocytochemistry

Cell cultures were fixed in 4% paraformaldehyde (Alfa Aesar, Ward Hill, MA) at 4^°^C for 20 minutes. Fixed cells were then permeated with 0.5% Triton X100 (Sigma-Aldrich) in PBS for 5min at room temperature. Next cells were incubated with specific antibodies (Abcam, Cambridge, UK) for pluripotency (Oct4, Nanog, Tra-1-81. Tra-1-60) and cardiac marker lineage (myosin heavy chain, connexin 43) at a 1:100 dilution, then in DyLight-conjugated secondary antibodies at a 1:500 dilution(Jackson ImmunoResearch Laboratories) and DAPI with VectaShield (Vector Laboratories, Burlingame, CA). The cell were imaged using an epifluorescence microscope (DMI 6000B, Lieca Microsystems, Wetzlar, DE).

### Cardiac differentiation

By adapting previously published protocols[10], AFSC derived iPSC were differentiated into cardiac cells by small molecule inhibition of the GSK3 and Wnt signaling pathways. Briefly, reprogrammed AFSC colonies were maintained on a feeder layer consisting of irradiated mouse embryonic fibroblast with daily changes of mTeSR1 media (Stem cell technologies, Vancouver, BC). Once sufficient cell numbers were obtained, undifferentiated colonies were dissociated in collagenase type 2 (Worthington Biochemical Corp., Lakewood, NJ) for 5 min then manually dislocated from the feeder layer, dispersed into single cell suspension, then plated as a monolayer of cells onto Matrigel (BD Biosciences, San Jose, CA) at a density of approximately 260,000 cells/cm2. Cells were expanded for 4 days in mTeSR1 media, which then corresponded to day 0. At this point, the media was changed to RPMI media with B27 supplement without insulin and the GSK3 inhibitor, CHIR99021, was exposed to the cells for 24 hours at a concentration of 12μM. At the end of 24 hours, media was replaced with fresh RPMI/B27 without insulin. At day 3, the Wnt inhibitor, IWP2, was added to RPMI/B27 without insulin at a concentration of 5μM. At day 7, insulin was added to the RPMI/B27 media. The occurrence of beating colonies was monitored through phase contrast microscopy after day 7.

### Flow cytometry

Cells were detached into suspension with Accutase (ThermoFisher) and stained with a fluorescently conjugated antibody for cardiac troponin T (BD Biosciences) with dilutions per manufacturer recommended concentrations. FACSDiva software (BD Biosciences) was used for all flow cytometry data collection. FlowJo software (Tree Star, Inc., Ashland, OR) was used for data analysis.

### Western blot

Western blot antibodies were purchased from Abcam Inc., electrophoresis and transfer materials were purchased from Bio-Rad (Hercules, CA), and developing materials were purchased from Li-Cor (Lincoln, NE). After 30 days of differentiation, total protein lysates were isolated from differentiated AFSC-iPSC and analyzed using a bicinchoninic acid kit (BCA; Thermo Scientific, Rockford, IL). Extracts were denatured using β-mercaptoethanol and boiling for 5 min, then diluted to equal concentrations of total protein. The samples were electrophoresed by 0.1% sodium dodecyl sulfate-polyacrylamide gel electrophoresis (SDS-PAGE) and blotted onto nitrocellulose membranes at 100 V for 1.5 and 1.0 hrs, respectively. Membranes were washed in tris-buffered saline with 0.05% Tween-20 (TBST), then blocked with Odyssey blocking buffer (Li-Cor) for 1 hour. Membranes were incubated overnight at 4°C with mouse monoclonal antibodies against MHC and cTnT (1:300 dilution in Odyssey blocker) and mouse monoclonal antibodies against GAPDH (1:1000 dilution in Odyssey blocker). Membranes were washed and submerged in IRDYE 800CW Goat anti-Mouse IgG secondary antibody with a dilution at 1:1000 in Odyssey blocker for 60min with gentle shaking. Membranes were washed and scanned using an Odyssey CLx scanner set to detect an 800nm wavelength. Western blots were normalized to GAPDH expression. Western blot analyze was performed using Image J (NIH, Bethesda, MD).

### Quantitative RT-PCR

Total RNA was extracted and purified using a PrepEase RNA spin kit (Affymetrix) and quantified by NanoDrop 1000 Spectrophotometer (Thermo Scientific). cDNA was synthesized from 2 μg of purified total RNA with random primers by Multiscribe Reverese Transcriptase (ThermoFisher). Quantitative real-time PCR was performed using the StepOnePlus system (ThermoFisher) and Veriquest Fast Probe qPCR Master Mix (Affymetrix), according to the manufacturer’s instructions. All data were normalized to mRNA level of housekeeping gene using the 2^ΔΔ^ method.

### Calcium and voltage transient analysis

The electrical behavior of the spontaneously contracting cells was measured through voltage-sensitive dye (Di-8-ANEPPS) and calcium-sensitive dye (Indo-1) with an epifluorescence microscope (Olympus, Center Valley, PA) and photomultiplier tubes detection system. Staining procedures for cells was the same for either dye. Cells were first washed with PBS warmed to 37oC to remove any serum contained in the media. Then 2μl of either Di-8-ANEPPS or Indo-1 at a concentration of 2mM was added to 2ml of Tyrode’s solution warmed to 37oC. The solution was then added to washed cells and allowed to sit at room temperature for 30min protecting the sample from light. The cells were then washed three times with fresh Tyrode’s solution and imaged using the epifluorescence microscope. For Indo-1 the detection wavelengths were 405nm and 485nm depending on the binding or calcium ions. Di-8-ANEPPS detection wavelengths were 560nm and 620nm depending of the shifts in membrane potentials. Emission data was collected and analyzed using Ion Optix software (Westwood, MA).

## Results

### Non-viral reprogramming of AFSC

**Fig 1:**
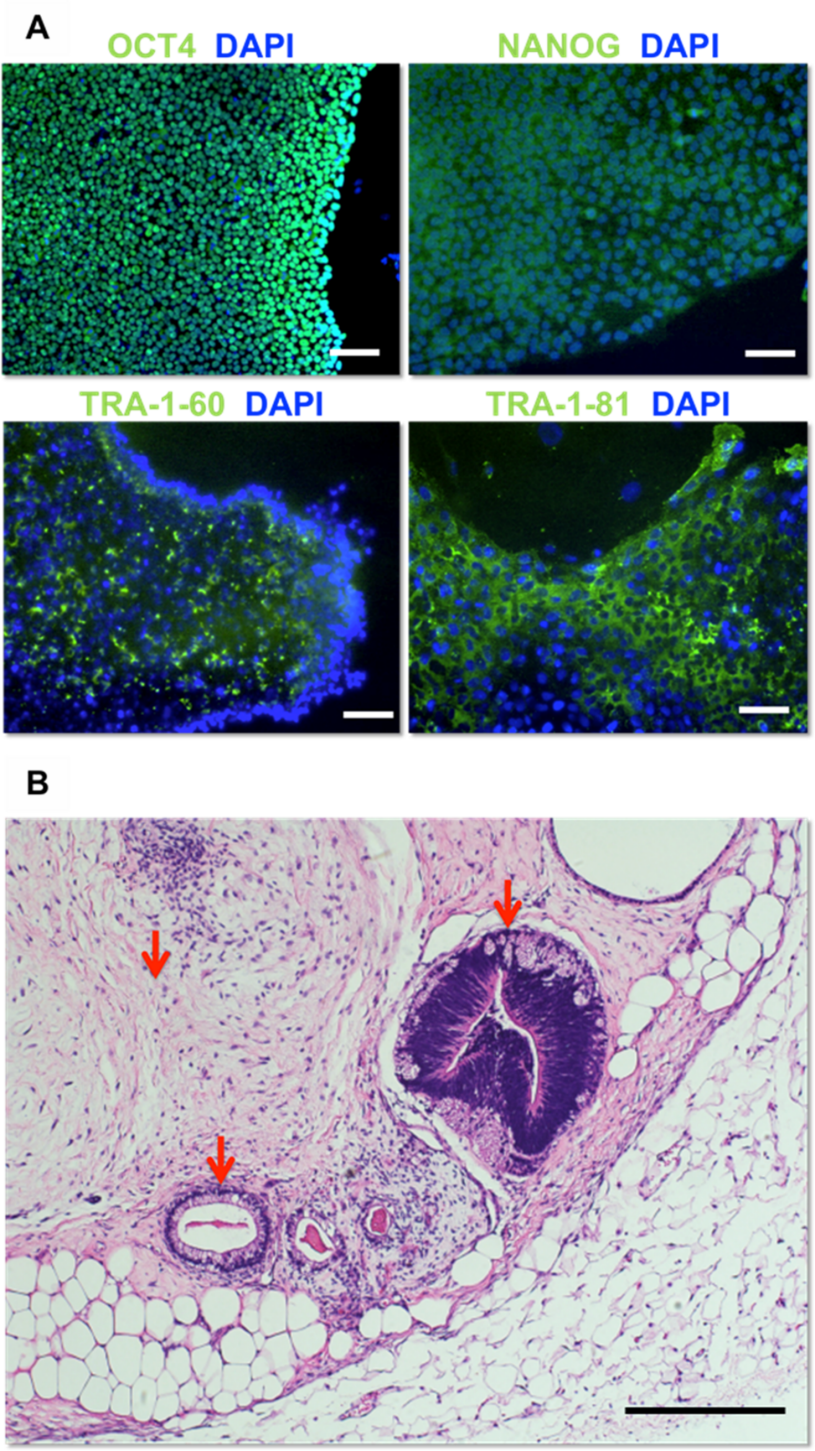
Pluripotent characterization of reprogrammed AFSC-iPSC. (A) Immunostaining for the human pluripotency markers OCT4, NANOG, TRA-1-60, and TRA-1-81. Scale bars, 100μm. (B) AFSC-iPSC-derived teratoma exhibiting neural epithelium (ectoderm, right) and gut epithelium (endoderm, bottom) surrounded by muscle and adipose tissue (mesoderm, top left) Scale bar, 100μm.

mRNA reprogramming of Passage 3 AFSC seeded at 2.6 x 10^4^ cells per cm^2^ yielded transfected and highly proliferative cells. Each 9.6cm^2^ well yielded approximately 10 colonies. Reprogrammed colonies were expanded 5–8 passages and then fixed and stained for pluripotent markers OCT4, NANOG, TRA-1-60, and TRA-1-81, all of which were expressed in all cells (Fig 1A). Cells were then subcutaneously injected into immunodeficient mice (NSG strain). Tumors formed after 8 to 12 weeks. Analysis by H&E staining exhibited tissue from mesodermal, ectodermal, and endodermal origin and confirmed pluripotency of injected AFSC iPSC (Fig 1B).

### Small molecule differentiation and genetic analysis of AFSC-iPSC derived CM

**Fig 2:**
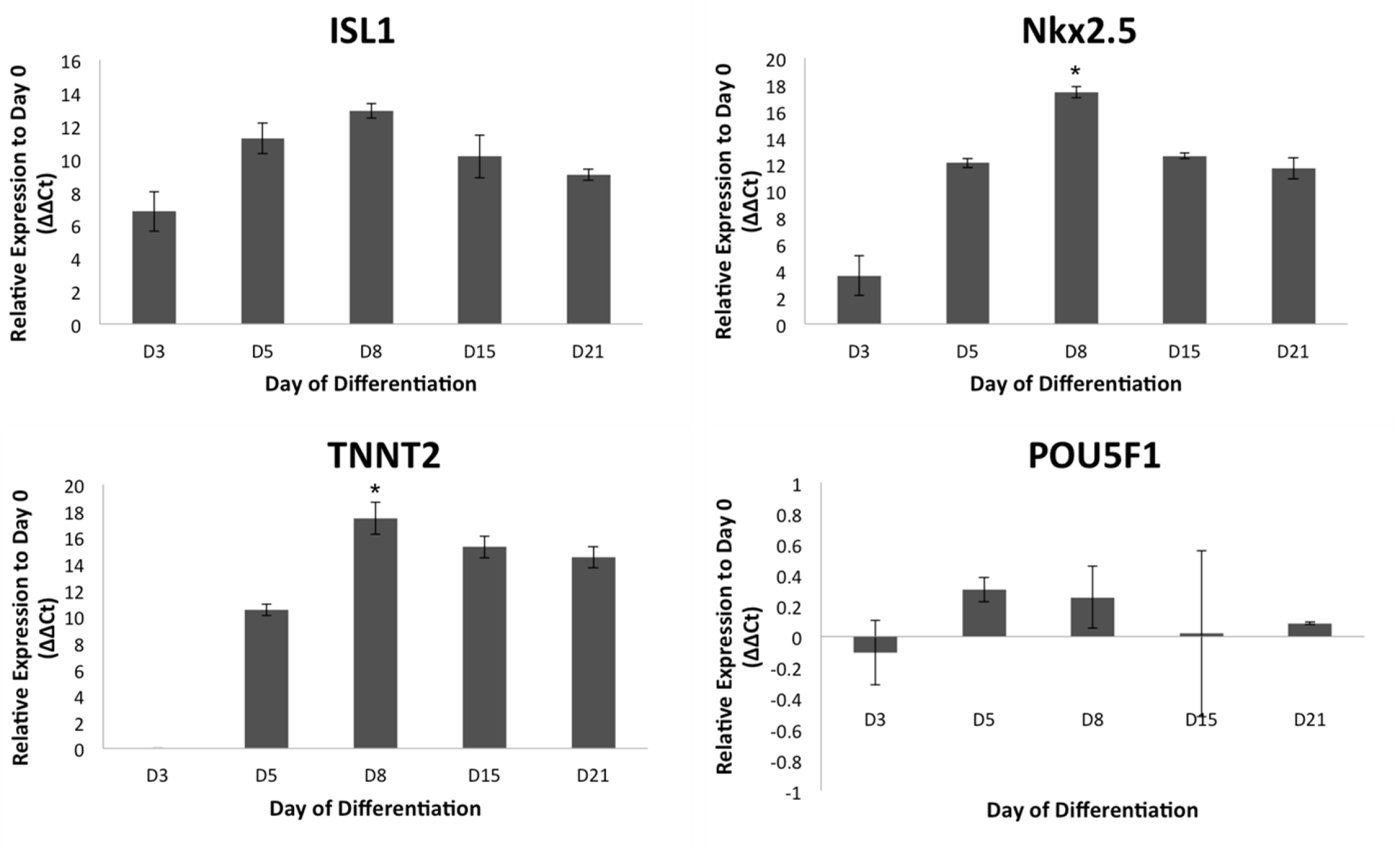
Genetic expression of cardiac differentiation markers. qRT-PCR assessment of genes for committed cardiac lineage (ISL1 and NKX2.5), cardiomyocytes (TNNT2), and pluripotency (POU5F1). Error bars represent standard deviations of mean values (n = 3, p<0.05, *significance compared to D0 other time points).

Genetic expression of ISL1, NKX2.5, TNNT2, and POU5F1 assessed at each time point was compared to expression at Day 0 (Fig 2). Upregulation occurred in cardiac progenitor genes ISL1 and NKX2.5 between Day 0 and Day 8 of differentiation and in late stage cardiac marker TNNT2 between Day 3 and Day 8 of differentiation. Among the days collected, peak expression was observed to be significant at Day 8 for NKX2.5 and TNNT2. Day 8, Day 15, and Day 21 data shows a decrease in expression of ISL1, NKX2.5, and TNNT2 between Day 8 and Day 15. No significant statistical difference was observed in the fold change in expression of POU5F1 between Day 3 and Day 21 of differentiation.

### Expression of late stage cardiac markers

**Fig 3:**
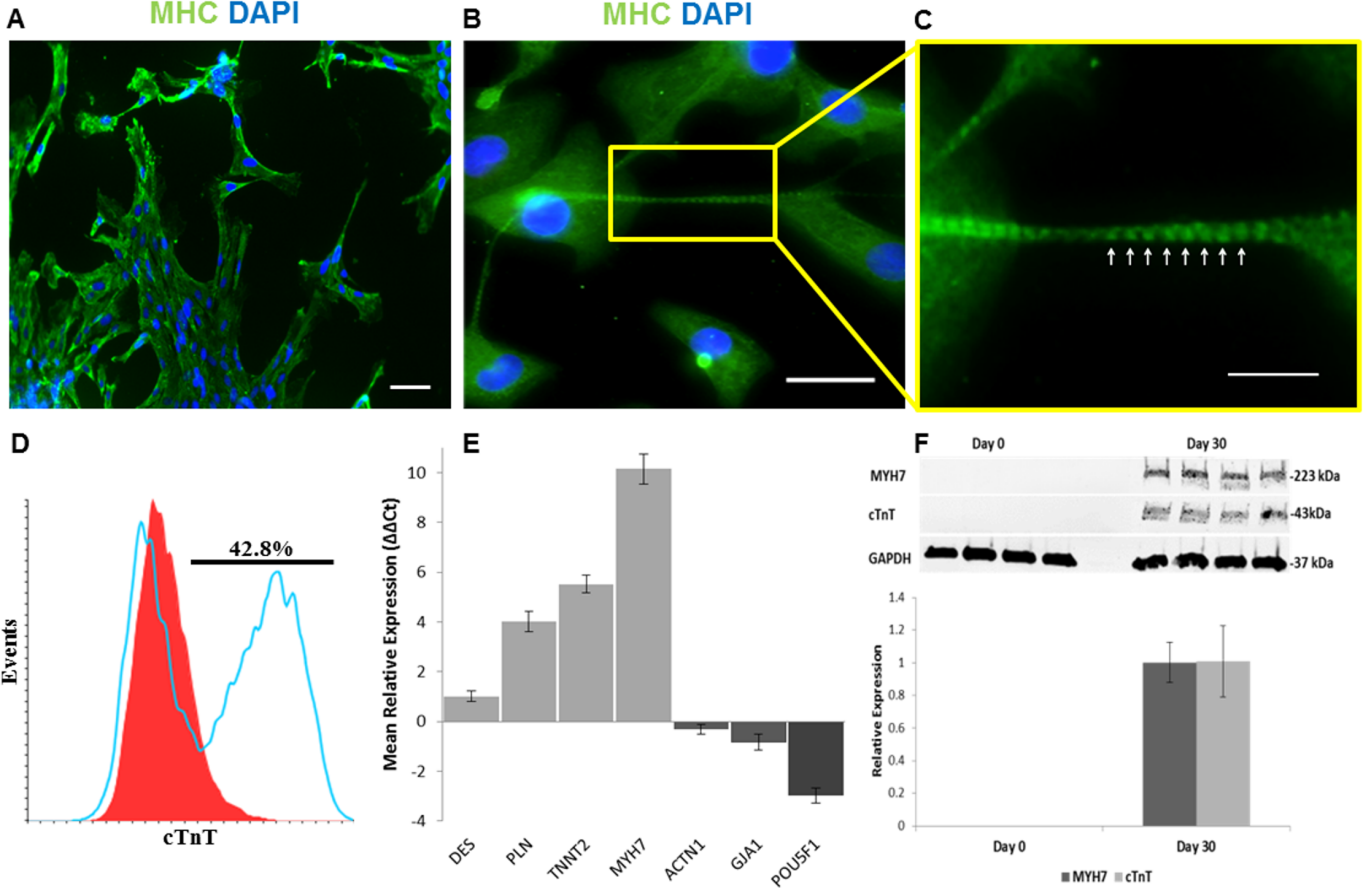
Characterization of spontaneously contracting CM cultures from AFSC-iPSC differentiated for 30 days. (A, B) Immunofluorescent staining of a contractile region for expression of sarcomere protein MHC. Scale bar, 20μm. (C) Magnified area shows striation pattern of sarcomeric cytoskeleton (white arrows). Scale bar, 10μm. (D) Day 15 cTnT expression analyzed by flow cytometry. (E) qRT-PCR assessment of upregulated cardiomyocyte genes DES, PLN, TNNT2, MYH7, and GJA1 and downregulated pluripotent gene POU5F1. (F) Western Blot analysis showing cardiac protein expression of cTnT and MHC.

Differentiated CM cultures were stained for visualization of MHC and Cx43. Immunohistochemical analysis shows contractile regions in Fig 3A and 3B. Higher magnification reveals a distinct repetitive banding pattern of MHC in cellular extensions, indicating immature sarcomeric cytoskeletal structure (Fig 3C). Connexin 43 was not organized but expression was cytoplasmic (S1 Fig). Differentiation efficiency was measured by flow cytometry expression of cTnT at Day 15. The average cTnT expression was showed to be 42.8 ± 12.3% (Fig 3D).

Gene analysis at Day 30 of differentiation by qRT-PCR showed significant upregulation of PLN, TNNT2, and MHC, genes for contractile machinery, and significant downregulation of pluripotent transcription factor POU5F1 compared to expression at Day 0 (Fig 3E). The human non-muscle alpha-actinin 1 was shown to be downregulated at Day 30. No significant difference was observed in expression of sarcomeric architecture regulator desmin and gap junction formation regulator Cx43, though spontaneous contraction in cultures was observed prior to collection. Protein analysis at Day 30 of differentiation by Western blot showed upregulation of cTnT and MHC, confirming the presence of both thin and thick filament contractile machinery (Fig 3F).

### Electrophysiology of contractile AFSC-iPSC derived CM

**Fig 4:**
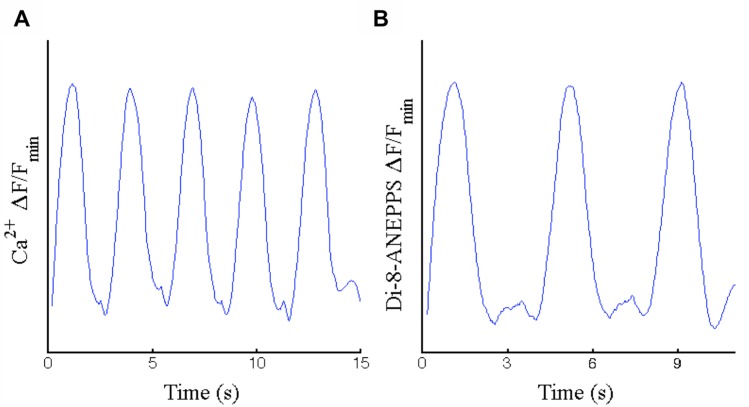
Waveforms generated from contracting regions as the change in ratio of fluorescence intensity readings over base-level fluorescence. (A) Calcium transient-based fluorescence using Indo1, and (B) Voltage transient-based fluorescence using Di-8-ANEPPS.

Spontaneously contracting CM cultures generated calcium and voltage fluorescent waveforms that demonstrate fluctuation in the ratio of extracellular to intracellular calcium (Fig 4A) and myocardial membrane depolarization (Fig 4B) upon contraction. Filtered and amplified fluorescent recordings had average periods of 3.04s (SD = 0.36, n = 5) and 3.83s (SD = 0.15, n = 3) for the different beating samples recorded in Fig 4A and 4B, respectively. Comparison shows a significant difference in beat frequency. In fact, a high degree of variability was observed in spontaneously contracting cultures subjected to identical differentiation, culturing, and environmental conditions.

## Discussion

Amniotic fluid is the ideal source of autologous cells for use in neonatal CHD treatment because of its capacity to be harvested during CHD diagnosis and the highly proliferative nature of AFSC [7] that enable the external development of tissue in parallel with gestation. Previous studies raise concerns of AFSC immune rejection upon transplantation however, because cells are induced to pluripotency, the antigenic profile is yet to be discovered but hypothesized to be of low immuno-stimulatory nature, similar to ESC and differentiated derivatives[15]. These experiments verify recent findings that iPSC can be generated from AFSC [12] and that AFSC-derived iPSC can be used to generate CM [16]. However, we are the first to demonstrate CM generation from AFSC without the use of viruses. Our studies support the feasibility of using amniotic fluid for CHD repair by showing that iPSC can be generated from AFSC using non-viral reprogramming, and that iPSC derived from AFSC non-virally are capable of cardiac differentiation. Previous studies from our group have verified that human AFSC express MSC markers CD29, CD44, CD73, CD90, and CD105, do not express endothelial marker CD31 or hematopoietic marker CD45, and express HLA-ABC but not HLA-DR[17]. The work of other investigators has shown that AFSC can be reprogrammed [11, 18] and that pluripotent cells derived from other sources can be differentiated to CM[4, 10, 19]. This study integrates these observations, while validating the use of a non-viral method of generating iPSC from AFSC and connecting the findings of others to demonstrate a direct link for differentiating CM from iPSC derived from AFSC.

While other well-established methods include the use of retroviruses, lentivirus, adenovirus, and Sendai virus as DNA delivery agents, mRNA delivery by lipofection was used for transfection to generate iPSC in this study. Lipofection delivery of mRNA is highly efficient, has no risk of transgene incorporation, and does not involve the use of viruses, reducing potential for complication in clinical translation[20]. This study is the first to demonstrate the use of lipofection for the transformation of AFSC to a pluripotent state. The achievement of pluripotency in this study, evident by expression of pluripotent markers OCT4, Tra-1-60, and Tra-1-81 *in vitro* and subcutaneous teratoma formation, is significant because it verifies the use of non-viral transfection. *in vitro* expression of NANOG supports dedifferentiation, though not maintenance of pluripotency[21, 22], while cytoplasmic localization is comparable to patterns in other studies[23].

The use of GSK3 and Wnt inhibition in this study validates cardiac differentiation for iPSC derived AFSC, but suggests a difference in its efficiency. Of the existing methods of differentiating pluripotent cells, GSK3 and Wnt inhibition has shown to produce the greatest yield efficiency of cardiomyocytes in monolayer culture [24]. However, these protocols were developed using embryonic stem cells (ESC) and iPSC derived from fibroblasts [25, 26]. It has been shown that the iPSC origin may influence the differentiation potential, specifically in regards to the efficiency and maturity [27]. In this study, spontaneous contraction in CM cultures differentiated from AFSC-iPSC was observed at approximately 21 days of differentiation, compared to 7–12 days as reported in other studies[4, 10, 19], though expression of ISL1, NKX2.5, and TNNT2 over the time course of differentiation resembles that of other studies [25, 28]. Cardiac immaturity is further supported by low upregulation of Desmin and indistinct intercalated disc gap junction formations marked by Cx43, in spite of clear cytoskeletal sarcomeric banding marked by MHC, obvious upregulation of cardiac encoding genes PLN, TNNT2, and MYH7, downregulation of pluripotent gene POU5F1, and transcription of cardiac machinery, evidenced by proteins cTnT and MHC. Alpha-actinin 1 is shown to have little to no activity in later stages of differentiation, eluding to the downregulation of non-muscular encoding genes. Immaturity is also evidenced by the recorded calcium and voltage-mediated fluorescence waveforms of contractile regions, exhibiting action potentials similar to immature CM because of the slow upstrokes during depolarization and the observed delayed after depolarizations [29], though these could also be explained by impedance mismatch from structural discontinuities. Delayed spontaneous contraction despite ordinary upregulation of NKX2.5 and ISL1, incompletely formed communication structures, and limited upregulation of the sarcomeric architecture regulator DES may imply a delayed developmental progression in the transcription and translation processes involved in the differentiation of CM from AFSC-iPSC using this protocol.

A major limitation during this study was the high degree of variability in contractile strength by day 30 of differentiation. Beating cultures were observed to become sparser, less frequent, weaker or stopped altogether. From this there can be speculation of decreasing viability of pacemaker cells or the growth of discontinuous structures due to death or fibrosis. Quantification of cTnT at Day 15 does show a substantial population of late stage cardiac cells however there is still variability in observed differentiation efficiency, delayed spontaneous contraction, and contractile viability. This study suggests that an alternative or modified differentiation procedure is needed for AFSC to be a viable cell source for deriving CM. Although this study is the first to use iPSC non-virally derived from AFSC, possible options for optimizing CM yield and differentiation consistency include inhibition timings, media components [25], and cell density.

## Conclusions

In the present study, AFSC were reprogrammed to iPSC by mRNA transfection, and AFSC-derived iPSC were differentiated into functional CM. Though differentiated CM were immature, as evidenced by delayed contraction, incomplete gap junction formation, and poor upregulation of Desmin, this study is the first to achieve functional CM from AFSC by non-viral means, as evidenced by sarcomere formation within cellular cytoskeleton, upregulation of NKX.2.5, ISL1, and cTnT, expression of cTnT and MHC, and clear calcium handling and membrane voltage propagation. In conclusion, while AFSC can now definitively be said to present a feasible source of functional CM generation, work is needed to improve differentiation efficiency and cardiac maturation.

## Supporting information

S1 Fig Immunofluorescent expression of connexin 43 in spontaneously contracting CM cultures from AFSC-iPSC differentiated for 30 days. Image overlay, from left to right: DAPI, Cx43, Combined. Scale bar, 20μm.
(TIFF)

S1 File Supporting information files. Datasets for qRT-PCR, calcium and voltage sensitive dyes.
(ZIP)
