# Supplementary figures and images for: Differentiation of spontaneously contracting cardiomyocytes from non-virally reprogrammed human amniotic fluid stem cells
Source: PLoS One. 2017 May 17;12(5):e0177824. doi: 10.1371/journal.pone.0177824 (PMC5435315; doi:10.1371/journal.pone.0177824)

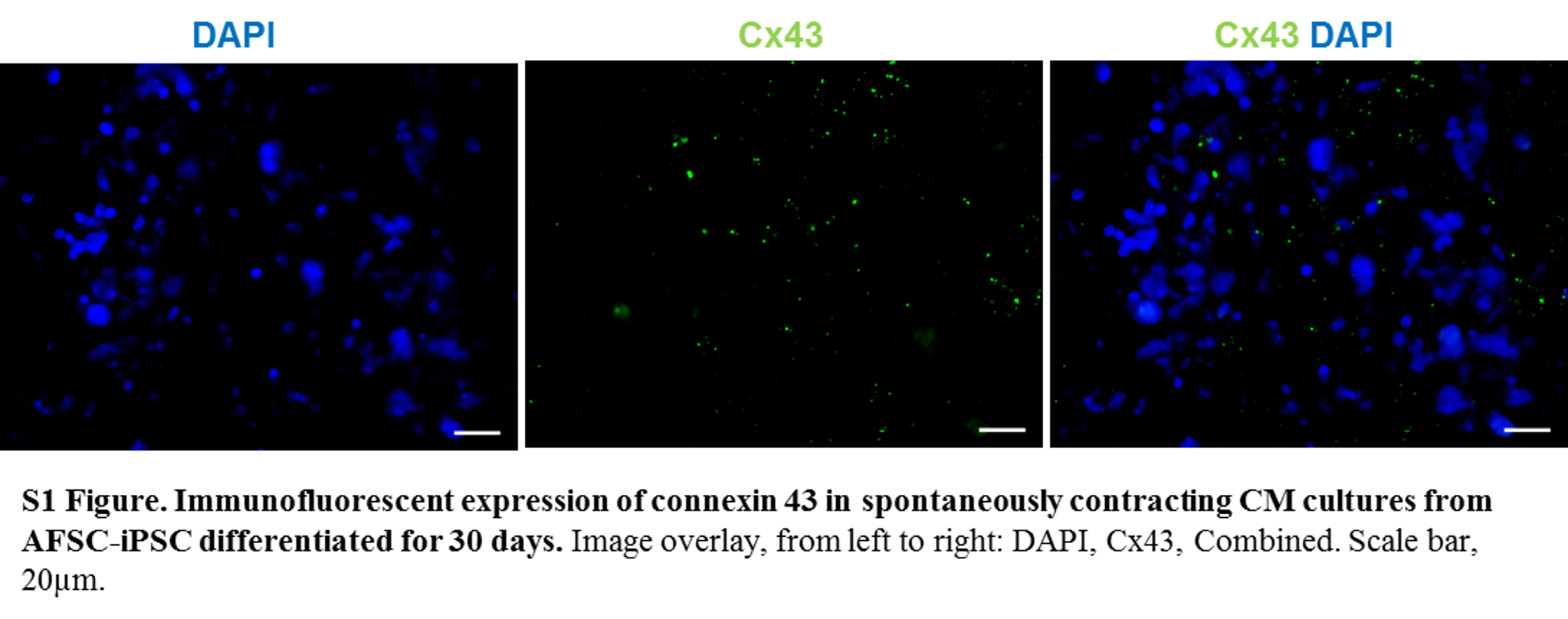

Supplement: S1 Fig — Image overlay, from left to right: DAPI, Cx43, Combined. Scale bar, 20μm. (TIFF) [file pone.0177824.s001.tiff]
